# Unique structure (construction and configuration) and evolution of the array of small serum protein genes of *Protobothrops flavoviridis* snake *

**DOI:** 10.1042/BSR20190560

**Published:** 2019-07-05

**Authors:** Takahito Chijiwa, Kento Inamaru, Ami Takeuchi, Marie Maeda, Kazuaki Yamaguchi, Hiroki Shibata, Shosaku Hattori, Naoko Oda-Ueda, Motonori Ohno

**Affiliations:** 1 Department of Applied Life Science, Faculty of Bioscience and Biotechnology, Sojo University, Kumamoto 860-0082, Japan; 2 Medical Institute of Bioregulation, Kyushu University, Fukuoka 812-8582, Japan; 3 Institute of Medical Science, University of Tokyo, Oshima-gun, Kagoshima 894-1531, Japan; 4 Department of Biochemistry, Faculty of Pharmaceutical Sciences, Sojo University, Kumamoto 860-0082, Japan

**Keywords:** accelerated evolution, genome structure, phospholipase A2, Protobothrops flavoviridis, serum, small serum protein, SSP gene array

## Abstract

The nucleotide sequence of *Protobothrops flavoviridis* (*Pf*) 30534 bp genome segment which contains genes encoding small serum proteins (SSPs) was deciphered. The genome segment contained five SSP genes (*PfSSPs*), *PfSSP-4, PfSSP-5, PfSSP-1, PfSSP-2*, and *PfSSP-3* in this order and had characteristic configuration and constructions of the particular nucleotide sequences inserted. Comparison between the configurations of the inserted chicken repeat-1 (CR1) fragments of *P. flavoviridis* and *Ophiophagus hannah* (*Oh*) showed that the nucleotide segment encompassing from *PfSSP-1* to *PfSSP-2* was inverted. The inactive form of *PfSSP-1*, named *PfSSP-1δ(Ψ)*, found in the intergenic region (I-Reg) between *PfSSP-5* and *PfSSP-1* had also been destroyed by insertions of the plural long interspersed nuclear elements (LINEs) and DNA transposons. The L2 LINE inserted into the third intron or the particular repetitive sequences inserted into the second intron structurally divided five *PfSSP*s into two subgroups, the Long SSP subgroup of *PfSSP-1, PfSSP-2* and *PfSSP-5* or the Short SSP subgroup of *PfSSP-3* and *PfSSP-4*. The mathematical analysis also showed that *PfSSP*s of the Long SSP subgroup evolved alternately in an accelerated and neutral manner, whereas those of the Short SSP subgroup evolved in an accelerated manner. Moreover, the ortholog analysis of *SSPs* of various snakes showed that the evolutionary emerging order of *SSP*s was as follows: *SSP-5, SSP-4, SSP-2, SSP-1*, and *SSP-3*. The unique interpretation about accelerated evolution and the novel idea that the transposable elements such as LINEs and DNA transposons are involved in maintaining the host genome besides its own transposition natures were proposed.

## Introduction

*Protobothrops flavoviridis* (*Pf*) (Crotalinae snake) [1] inhabit the southwestern islands of Japan, mainly, Amami-Oshima, Tokunoshima, and Okinawa. The venom of *P. flavoviridis* contains a huge variety of toxic proteins. The representatives are snake venom metalloproteases (SVMPs) [2], serine proteases [3], phospholipase A_2_s (PLA_2_s) [4–7], and triflin [8]. High molecular weight SVMPs, called HR1a and HR1b [9], and middle molecular weight SVMPs, called HR2a and HR2b [10], are isozymes of each other and cause severe hypodermic hemorrhage [11]. Low molecular weight SVMP, called HV1, induces apoptosis of vascular endothelial cells [12]. Serine protease, called Flavoxobin [13,14], is known as coagulant factor. Triflin is a neurotoxin-like protein and blocks muscle contraction [15]. *Protobothrops* genus snake venom PLA_2_ isozymes, which hydrolyze phospholipids [16,17], are generally classified into four groups, according to the primary structures and physiological activities [18]: that is, neutral [Asp^49^]PLA_2_, named PLA2 (pI 7.9, highly lipolytic and myolytic) [19,20]; basic [Asp^49^]PLA_2_, named PLA-B (pI 8.6, edema-inducing) [21]; highly basic [Asp^49^]PLA_2_, named PLA-N (pI 10.3, neurotoxic) [22]; and two [Lys^49^]PLA_2_s, named BPI and BPII (pIs 10.2 and 10.3, both weakly lipolytic but strongly myolytic) [19,23,24].

On the other hand, *P. flavoviridis* serum is known to contain inhibitory proteins to neutralize their venomous activities. Such serum proteins are thought to act defensively on occasions of the accidental bites by fellow snakes. PLA_2_ inhibitors (PLIs) which suppress snake venomous PLA_2_ activity [25,26], Habu serum factor (HSF) which inhibits the hemorrhagic induced by the SVMPs [27,28], and small serum proteins (SSPs) [29] are such well-known proteins. SSPs are the low molecular weight (∼10 kDa) serum proteins and the five homologs, named *Pf*SSP-1, -2, -3, -4, and *Pf*SSP-5, have been found from *P. flavoviridis* sera [30]. *Pf*SSPs form complexes with HSF in *P. flavoviridis* serum [8] and have various counterparts of *P. flavoviridis* venom proteins; *Pf*SSP-2 and *Pf*SSP-5 show high affinity to triflin [8], *Pf*SSP-1 and *Pf*SSP-4 to HV1 [31], and *Pf*SSP-3 to Flavorase, non-hemorrhagic SVMP [29]. Recently, we found that the homolog of *Pf*SSP-2 binds to BPII [32].

The complementary DNA (cDNA)s encoding five *Pf*SSPs, were isolated and the nucleotide sequences of the cDNAs were also determined [8]. It should be noted that the cDNAs encoding *Pf*SSP-3 or *Pf*SSP-4 are interrupted by nonsense mutations at the same site of the fourth exon and express the truncated mature protein. The genome fragment, in which the genes encoding *Pf*SSP-1 and *Pf*SSP-2, designated as *PfSSP-1* and *PfSSP-2*, respectively, were arranged in tandem, was also cloned. The nucleotide sequence of the genome fragment from *PfSSP-1* to *PfSSP-2* including the intergenic region (I-Reg) between *PfSSP-1* and *PfSSP-2*, named as *Pf*I-Reg12 (in the present paper), was determined [33]. Mathematical analysis of the nucleotide sequences of the two genes showed that they have evolved in an accelerated manner to acquire the different amino acid sequences [8,33].

In the present study, 30534 bp of *P. flavoviridis* genome segment containing five *PfSSP*s; *PfSSP-4, PfSSP-5, PfSSP-1, PfSSP-2*, and *PfSSP-3* in this order, was deciphered. The particular nucleotide sequences of the long interspersed nuclear elements (LINEs), the DNA transposons, and the repetitive sequences were identified in the introns of *PfSSP*s and the I-Regs of the array of *PfSSP*s. The comparison analysis of the configuration of the fragments of chicken repeat-1 (CR1) LINE between *P. flavoviridis* and *Ophiophagus hannah* (*Oh*) (Elapidae snake) [34] showed that the chromosome inversion of the genome segment encompassing from *PfSSP-1* to *PfSSP-2* occurred and the inactive form of another *PfSSP-1, PfSSP-1δ(Ψ)*, was formed at that site. Moreover, according to the types of the nucleotide sequences inserted into the intron of the gene, five *PfSSP*s were divided into two subgroups. The Long SSP subgroup, which consisted of *PfSSP-1, PfSSP-2*, and *PfSSP-5*, contained the large fragment of L2 LINE in the third intron of the gene. The Short SSP subgroup, which consisted of *PfSSP-3* and *PfSSP-4*, contained the particular repetitive sequences in the second intron of the gene and no L2 LINE in the third intron. The mathematical analysis of the nucleotide sequences of the genes also showed that *PfSSP*s of the Short SSP subgroup evolved in an accelerated manner, whereas those of the Long SSP subgroup evolved alternately in an accelerated and neutral manner. The ortholog analysis of various snake *SSP*s suggested that the evolutionary emerging order of *PfSSP*s was reflected in the order of their configuration on the chromosome. In addition to the unique interpretation about accelerated evolution, a novel idea was proposed that the transposable elements such as LINEs and DNA transposons are involved in maintaining the host genome besides their transposition natures.

## Experimental

### Materials

*P. flavoviridis* specimen of Amami-Oshima island was provided from the Institute of Medical Sciences of the University of Tokyo. High molecular weight genomic DNAs were prepared from the liver of the snake according to the method of Blin and Stafford [35]. Restriction endonucleases and KOD plus DNA polymerase were purchased from Nippon Gene (Tokyo, Japan) and TOYOBO (Osaka, Japan), respectively. The other reagents and antibiotics were from Nacalai Tesque (Kyoto, Japan) and Takara Bio (Shiga, Japan). Specific oligonucleotide primers were synthesized by GENNET (Fukuoka, Japan) (Table 1).

**Table 1:** The primers utilized for acquiring the nucleotide sequence of the genome domain of the array of *PfSSP*s

Name	Positions	Nucleotide sequence (GC content: %, Tm: °C)
JUS1	6973-6993(f)	5′-ATT CCT CCC TAC CAA gAg TCT-3′ (47, 62)
JUS2	11016-11037(f)	5′-TCT ATg TgA Agg gAT gAg AAT C-3′ (40, 62)
JUS5	10961-10983(r)	5′-CAT gCC AAC ATg AAT CCT ATA gg-3′ (43, 64)
JUS8	13451-13473(f)	5′-ACC CAC Tgg AAT AAA TTT CTC AT-3′ (35, 62)
TOY1	1098-1121(f)	5′-ggA gTA TTC CTT TAC CTg AAA Tgg-3′ (47, 68)
TOY2	6964-6985(r)	5′-ggT Agg gAg gAA TTA CCg ggA g-3′ (59, 70)
TOY6	-220-198(f)	5′-ggC TgC ACA TCT ggC TgT TTC AA -3′ (47, 68)
TOY7	1180-1201(r)	5′-TTC CTC CTg gCA gTg TTA gAC C -3′ (45, 68)
prs-1	29119-29138(r)	5′-gAg TgT TCC TCT ACC TAT Ag-3′ (45, 58)
prs-2	26172-26192(f)	5′-TTg TCA TTC TCT gAg AAg Tgg-3′ (43, 60)
prs-5	26342-26364(r)	5′-CgC TTg CAC TgA AgA TgC AAT gg-3′ (52, 70)
prs-6	22895-22917(f)	5′-AAg AgC AgC ACC TCT CTg TgA Ag-3′ (52, 70)
prs-13	30295-30314(r)	5′-TTC CTT CTg gCA gTg gAT TC-3′ (50, 60)
prs-14	29044-29066(f)	5′-TTC TCC Tgg CgT TAT TAg ACA-3′ (43, 60)

The f and r in the parentheses after position numbers indicate the direction of the primers: ‘f, forward’ or ‘r, reverse’ means whether the directions of elongations are same or opposite to those of transcriptions, respectively. The nucleotide positions were referenced to the nucleotide sequence reported in the present study (MK574076).

### Determination of the nucleotide sequence of *P. flavoviridis* genome segment containing *PfSSPs*

From the BLAST analysis against HabAm1 (Habu Amami version 1) [36], we found that *PfSSP-5, PfSSP-1, PfSSP-2*, and *PfSSP-3* are harbored in this order on the scaffold 2858. Furthermore, *PfSSP-4*, which had not been identified via gene prediction so far, was also found in the 5′ upstream region of this arrangement of four *PfSSP*s on the scaffold 2858. In order to acquire the complete nucleotide sequence of this genome segment, genomic polymerase chain reactions (PCRs) against *P. flavoviridis* genome were performed with the specific sense and antisense primers referring to the nucleotide sequences of transcripts and genes of *Pf*SSP or HabAm1 (Table 1).

The sense primer, JUS1, 5′-ATT CCT CCC TAC CAA gAg TCT-3′, which can anneal specifically to the first exon of *PfSSP-5*, and the antisense primer, JUS2, 5′-TCT ATg TgA Agg gAT gAg AAT C-3′, which can anneal specifically to the fourth exon of *PfSSP-5*, referring to the nucleotide sequence of the cDNA encoding *Pf*SSP-5 (AB360910), amplified the 4065-bp genome fragment, named *Pf*jb-I. The *Pf*jb-I fragment was cloned into pCR®-Blunt II-TOPO® vector (Life Technologies, Carlsbad, CA, U.S.A.) and transformed with DH5α competent cells (Takara Bio) and sequenced. The nucleotide sequences were determined with an ABI 3130xl capillary sequencer. The *Pf*jb-I was found to encompass from the first exon to the fourth exon of *PfSSP-5*. In the present study, *PfSSP-5* contained extra Val at position 89 encoded by ^265^GTG and Asp at position 91 encoded by ^271^GAT was also substituted to Asn encoded by ^271^AAT.

In order to acquire the nucleotide sequence of the I-Reg between *PfSSP-5* and *PfSSP-1*, named as *Pf*I-Reg51, genomic PCR was carried out against *P. flavoviridis* genome with the sense primer, JUS5, 5′-CAT gCC AAC ATg AAT CCT ATA gg 3′, which can anneal to the fourth exon of *PfSSP-5*, and the antisense primer, JUS8, 5′-ACC CAC Tgg AAT AAA TTT CTC AT-3′, which can anneal to the fourth exon of *PfSSP-1*, referring to the nucleotide sequence of SSP-1 gene (AB769881), amplified the 2513 bp genome fragment, named *Pf*jb-II. The *Pf*jb-II fragment was also cloned and sequenced. The *Pf*jb-II was found to encompass from the fourth exon of *PfSSP-5* to the fourth exon of *PfSSP-1* including *Pf*I-Reg51. The 4065 bp *Pf*jb-I overlapped 77 bp with the 2513 bp *Pf*jb-II. The physical structure of 6501 bp segment encompassing from the fourth exon of *PfSSP-5* to the first exon of *PfSSP-1* was deciphered.

As the nucleotide sequences of the first exons of *PfSSP-4* and *PfSSP-3* are completely identical, it is hard to design the specific primer at the first exon which can amplify each gene differentially. So, the sense primer, TOY6, 5′-ggC TgC ACA TCT ggC TgT TTC AA-3′, which can anneal specifically to 220 bp 5′ upstream of the first exon of *PfSSP-4*, and the antisense primer, TOY7, 5′-TTC CTC CTg gCA gTg TTA gAC C-3′, which can anneal specifically to the second intron of *PfSSP-4*, avoiding the nucleotide sequence of the open reading frame (ORF) of *PfSSP-4* (AB360909), amplified the 1421 bp genome fragment, named *Pf*jb-IV. The *Pf*jb-IV fragment was cloned and sequenced. The *Pf*jb-IV was found to encompass from 220 bp 5′ upstream of the first exon to the second intron of *PfSSP-4*.

Then, the sense primer, TOY1, 5′-ggA gTA TTC CTT TAC CTg AAA Tgg-3′, which can anneal specifically to the second exon of *PfSSP-4*, and the antisense primer, TOY2, 5′-ggT Agg gAg gAA TTA CCg ggA g-3′, which can anneal specifically to the first exon of *PfSSP-5*, referring to the nucleotide sequence of the cDNA encoding *Pf*SSP-5 (AB360910), amplified the 5888 bp genome fragment, named *Pf*ib-III. The *Pf*jb-III fragment was cloned and sequenced. The *Pf*jb-III was found to encompass from the second exon of *PfSSP-4* to the first exon of *PfSSP-5* including the I-Reg between *PfSSP-4* and *PfSSP-5*, named as *Pf*I-Reg45.

The 5888 bp *Pf*jb-III overlapped 104 bp with the 1421 bp *Pf*jb-IV. The physical structure of 7205 bp segment encompassing from 220 bp 5′ upstream of the first exon of *PfSSP-4* to the first exon of *PfSSP-5* was deciphered. The nucleotide sequence of the genome segment containing from *PfSSP-1* to *PfSSP-2* has been already reported by Tanaka et al. ([33], AB769881). Moreover, the 5888 bp *Pf*jb-III overlapped 13 bp with the 4065 bp *Pf*jb-I. The physical structure of 23202 bp segment encompassing from 220 bp 5′ upstream of the first exon of *PfSSP-4* to 5′ terminal of the first exon of *PfSSP-2*, including the nucleotide sequence of the genome segment encompassing from *PfSSP-1* to *PfSSP-2*, was deciphered.

The sense primer, prs-2, 5′-TTg TCA TTC TCT gAg AAg Tgg-3′, which can anneal specifically to 571 bp 3′ downstream of the fourth exon of *PfSSP-3*, and the antisense primer, prs-1, 5′-gAg TgT TCC TCT ACC TAT Ag-3′, which can anneal specifically to the second exon of *PfSSP-3*, referring to the nucleotide sequence of the cDNA encoding *Pf*SSP-3 (AB360908), amplified the 2967 bp genome fragment, named P*f*jb-V. The *Pf*jb-V fragment was cloned and sequenced. The *Pf*jb-V was found to encompass from 571 bp 3′ downstream of the fourth exon to the second exon of *PfSSP-3*.

Then, the sense primer, prs-6, 5′-AAg AgC AgC ACC TCT CTg TgA Ag-3′, which can anneal specifically to the first exon of *SSP-2*, and the antisense primer, prs-5, 5′-CgC TTg CAC TgA AgA TgC AAT gg-3′, which can anneal specifically to 378 bp 3′ downstream of the fourth exon of *PfSSP-3*, avoiding the nucleotide sequence of the ORF of *PfSSP-3* (AB360908), amplified the 3470 bp genome fragment, named P*f*jb-VI. The *Pf*jb-VI fragment was cloned and sequenced. The *Pf*jb-VI was found to encompass from 401 bp 5′ upstream of the fourth exon of *PfSSP-3* to the first exon of *PfSSP-2*. The 2967 bp P*f*jb-V overlapped 193 bp with the 3470 bp *Pf*jb-VI.

The sense primer, prs-14, 5′-TTC TCC Tgg CgT TAT TAg ACA-3′, which can anneal specifically to the second intron of *PfSSP-3*, and the antisense primer, prs-13, 5′-TTC CTT CTg gCA gTg gAT TC-3′, which can anneal specifically to 59 bp (the present study) 5′ upstream of the first exon referring to the nucleotide sequence of *PfSSP-3* (AB360908), amplified the 1269 bp genome fragment, named P*f*jb-VII. The *Pf*jb-VII fragment was cloned and sequenced. The *Pf*jb-VII was found to encompass from the second intron to 59 bp 5′ upstream of the first exon of *PfSSP-3*. The 1269 bp *Pf*jb-VII overlapped 93 bp with the 2967 bp *Pf*jb-V. The physical structure of 7420 bp segment encompassing from the first exon of *PfSSP-2* to 59 bp 5′ upstream of the first exon of *PfSSP-3* was deciphered. The 3470 bp *Pf*jb-VI overlapped 37 bp with the first exon of *PfSSP-2* (AB769881). Finally, the physical structure of 30534 bp segment encompassing from 220 bp 5′ upstream of the first exon of *PfSSP-4* to 59 bp 5′ upstream of the first exon of *PfSSP-3* was completely deciphered.

The nucleotide sequence and the detailed annotations of the genome domain composed of *Pf*jb-IV, *Pf*jb-III, *Pf*jb-I, *Pf*jb-II, *Pf*jb-VI, *Pf*jb-V and *Pf*jb-VII, are available in the Genbank/EMBL/DDBJ databases under accession number MK574076.

### Determination of the nucleotide sequences and the chromosomal configurations of the genes encoding the orthologs of *PfSSP*s of various snakes

The draft nucleotide sequences of *O. hannah* [34,37], *Python bivittatus* (Pythonidae snake) [38,39], *P. mucrosquamatus* (Viperidae snake) [40,41], and *Thamnophis sirtalis* (Colubridae, Naticinae snake) [42,43], were downloaded to make personal genome databases. Referring to the nucleotide sequences and the deduced amino acid sequences of *PfSSP*s via tblastn or blastn, the nucleotide sequences encoding the orthologs of *PfSSP*s and the flanking regions of them in each snake genome data were deciphered.

### RepeatMasker analysis of the nucleotide sequence of the genome segment harboring *SSP*s

The personal database was constructed with the repetitive sequences of the genomes of various organisms collected from the Repbase of the Genetic Information Research Institute [44]. RepeatMasker was carried out the nucleotide sequences of the genome segments containing *SSP*s of *O. hannah, P. bivittatus, P. flavoviridis, P. mucrosquamatus*, and *T. sirtalis*, against the database via BLAST+, RMBlast (NCBI), and Tandem Repeats Finder (Boston University) [45].

### Mathematical analysis

Alignment of the amino acid sequences of *Pf*SSPs was performed using ClustalX software. The nucleotide sequences of ORFs encoding the mature proteins of *Pf*SSPs were rearranged, removing the gaps, by PAL2NAL according to the aligned amino acid sequences. The rates of synonymous (*K*_S_) and nonsynonymous (*K*_A_) substitutions per site between the ORFs of the genes were calculated using Nei-Gojobori method as implemented with PAML [46]. The rates of substitutions of the introns (*K*_N_) were calculated from the aligned sequence data.

## Results and discussion

### Peculiar structure of the array of *PfSSP*s

**Figure 1:**
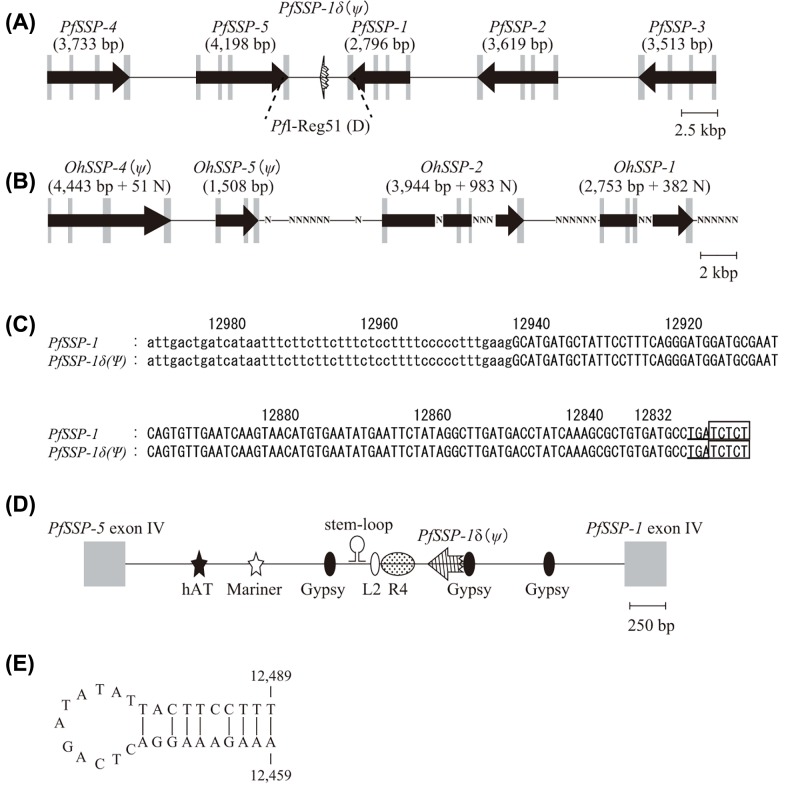
Detailed analysis of the genome segments of *P. flavoviridis* and *O. hannah* containing *SSP*s (**A**)** The schematic representation of *P. flavoviridis* 30534 bp genome segment containing the array of six *PfSSP*s including the fragmented *PfSSP-1, PfSSP-1****δ*(*Ψ*). Bold and hatched broken arrows indicate the areas and the directions of the transcription of the genes in the segment. Gray bars represent exons. (**B**)** The schematic representation of *O. hannah* 17678 bp (except for 4745 ‘N’s) genome fragment containing the array of four *OhSSP*s.** ‘N’ means the unidentified nucleotides. (**C**)** The alignment of the nucleotide sequences of *PfSSP-1δ*(*Ψ*) and the corresponding portion of *PfSSP-1*.** The portions of introns 3 are shown in lower case letters and those of exons 4 are shown in upper case letters. The stop codons are underlined. 3′ UTRs of exons 4 are enclosed in the squares. The numerals above the sequences are the position numbers of the corresponding nucleotides reported in the present study (MK574076). (**D**)** The schematic configuration of the fragments of LINEs and DNA transposons inserted into the I-Reg *Pf*I-Reg51.** Three closed ellipses, open ellipse, and specked ellipse represent the inserted fragments of LINEs, Gypsy, L2, and R4, respectively. Closed and open stars represent the inserted DNA transposons, hAT and Mariner, respectively. The position of the stem-loop structure is also shown. *PfSSP-1δ(Ψ)* is indicated by the hatched arrow. (**E**)** The predicted stem-loop structure of the 30 nucleotides located between the fragment of Gypsy and the L2 fragment-R4 fragment-*PfSSP-1(****Ψ****)* arrangement.** The secondary structure is deduced based on the nucleotide sequence by RNA secondary structure prediction of GENETYX ver. 16. The numerals at both termini of the sequence are the position numbers of the corresponding nucleotides reported in the present study (MK574076). Abbreviation: UTR, untranslated region.

The nucleotide sequence of 30534 bp of *P. flavoviridis* genome segment was deciphered as described above and found to contain the array of five *PfSSP*s, that is, *PfSSP-4, PfSSP-5, PfSSP-1, PfSSP-2*, and *PfSSP-3* in this order (Figure 1A). The precise construction of each of the five *PfSSP*s including the promoter and four exons was revealed (MK574076) 3733 bp of *PfSSP-4*, 4198 bp of *PfSSP-5*, 2796 bp of *PfSSP-1*, 3619 bp of *PfSSP-2*, and 3513 bp of *PfSSP-3*.

The draft genome data of *O. hannah* (Elapidae snake) was also investigated to decipher the nucleotide sequences of the genome segment harboring the orthologs of *PfSSP*s (see the details in the ‘Determination of the nucleotide sequences and the chromosomal configurations of 221 the genes encoding the orthologs of PfSSPs of various snakes’ subsection of ‘Experimental’ section). Then, four nucleotide sequences of the genes which encode SSP-1, SSP-2, SSP-4, and SSP-5, were identified. As the draft genome data contained many unidentified nucleotides which were described as ‘N’, only the nucleotide sequences of four exons of the gene encoding SSP-4 were identified. The nucleotide sequences of the third exons of the genes encoding SSP-1 and SSP-2 were partly identified, and that of the fourth exon of the gene encoding SSP-5 could not be identified (Figure 1B). The nucleotide sequences encoding SSP-1 and SSP-2 were named as *OhSSP-1* and *OhSSP-2*, respectively. As the nucleotide sequences of the third exons of both genes encoding SSP-4 and SSP-5 contained the insertion of 98 and 8 nucleotides to cause nonsense mutation, they were named as *OhSSP-5(Ψ)* and *OhSSP-4(Ψ)*, respectively. It should be noted that the directions of the transcription of *OhSSP*s were all the same, in contrast with those of *PfSSP-4* and *PfSSP-5* were opposite to those of the other three *PfSSP*s, *PfSSP-1, PfSSP-2*, and *PfSSP-3*. It is likely that the genome fragment harboring *PfSSP-1, PfSSP-2*, and *PfSSP-3*, has been inverted.

### Complicated construction of the I-Reg between *PfSSP-5* and *PfSSP-1*

Detailed analysis showed that the I-Reg between *PfSSP-5* and *PfSSP-1, Pf*I-Reg51, was an interesting structure. First, the nucleotide sequence which encodes another fragmented *PfSSP-1* was found in the middle portion of *Pf*I-Reg51. The nucleotide sequence, named *PfSSP-1δ(Ψ)*, consisted of 48 bp of the 3′ portion of the third intron and the fourth exon with five nucleotides of the 3′ untranslated region (UTR) of *PfSSP-1* (Figure 1C). Second, the Repeatmasker revealed that the fragments of three types of LINE, L2, R4, and Gypsy, were inserted so as to sandwich the *PfSSP-1δ(Ψ)* (Figure 1D). The two fragments of L2 and R4 LINEs were located in the 3′ downstream of *PfSSP-1δ(Ψ)* and that of Gypsy LINE was located in the 5′ upstream of *PfSSP-1δ(Ψ)*. Each of the three fragments encoded most of the reverse transcriptase (RT) domain of each LINE.

Two DNA transposon fragments hAT [47], Mariner [48], and another Gypsy LINE fragment were inserted in the region between *PfSSP-5* and the L2 fragment-R4 fragment-*PfSSP-1δ(Ψ)* arrangement. Both DNA transposons are known to carry out gene conversion via double-strand break [49,50]. In addition, the 30 bp nucleotide sequence predicted to form the stem-loop structure (Figure 1E) was found immediately next to the L2 fragment-R4 fragment-*PfSSP-1δ(Ψ)* arrangement. The stem-loop structure is also known to be the scaffolding of the gene conversion [51,52]. *PfSSP-1δ(Ψ)* should be the remnant of the amplified *PfSSP-1* which was destroyed by the plural times of insertions of LINEs and DNA transposons after being amplified into *Pf*I-Reg51.

### Chromosome inversion interrupted the array of *PfSSP*s

**Figure 2:**
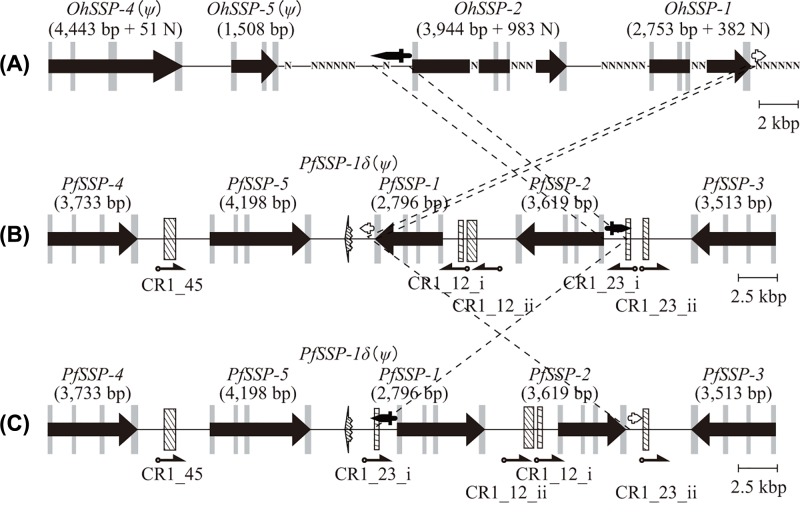
Comparison of the genome segments of *P. flavoviridis* and *O. hannah* containing *SSP*s Comparison of the schematic configuration of (**A**) the array of *OhSSP*s, (**B**) the array of *PfSSP*s, and (**C**) the temporary array of *PfSSP*s in the case where the chromosome inversion did not occur. White and black daggers represent the positions and the directions of the nucleotide sequences of α and *β*, which are linked with dashed lines to each other. Harpoons represent the positions and the directions of the transcription of the inserted fragments of CR1 LINE.

Further investigation of the nucleotide sequences of the arrays of *SSP*s of *P. flavoviridis* and *O. hannah* showed that there were two pairs of the particular nucleotide sequences. One pair was 140 nucleotides in the 3′ downstream of *PfSSP-1* and 140 nucleotides in the 3′ downstream of *OhSSP-1*, the other pair was 937 nucleotides in the 5′ upstream of *PfSSP-2* and 961 nucleotides in the 5′ upstream of *OhSSP-2* (Figure 2A,B). The nucleotide sequence of the former pair was designated as ‘α’ and that of the latter pair as ‘β’. The identity between the nucleotide sequences of α or β pairs was 69 or 65%, respectively, but the direction of the nucleotide sequences of the α or *β* pairs was opposite. These findings showed that the *P. flavoviridis* genome segment encompassing from the α sequence to the *β* sequence had been inverted, moreover the tandem arrangement of *PfSSP-1* and *PfSSP-2* had already been formed before the inversion occurred.

Interestingly, RepeatMasker analysis also showed the five fragments of CR1 LINE, which is the most major LINE contained in the reptilian genome, were found in all the *Pf*I-Regs except for *Pf*I-Reg51. They are CR1_45, the CR1 fragment inserted in the *Pf*I-Reg45, and CR1_12_i and CR1_12_ii, or CR1_23_i and CR1_23_ii, the CR1 fragments inserted in this order at the middle portion of *Pf*I-Reg12 or *Pf*I-Reg23, respectively (Figure 2B). CR1 is composed of two ORFs, ORF1 and ORF2 [53]. ORF1 encodes RNA binding protein and ORF2 encodes two-domain protein which is composed of endonuclease (EN) and RT domains. The RT domain of CR1 consists of ten subdomains from 0 to IX and a carboxy-terminal conserved region (CTCR), which is known to be the scaffold of reverse-transcription of CR1 LINE [54–56]. The fragments CR1_45 and CR1_12_ii contained four subdomains from III to VI and from IV to VII of the RT domain, respectively. On the other hand, each of the fragments CR1_12_i, CR1_23_i, and CR1_23_ii contained only the CTCR of the RT domain. Interestingly, the direction of the transcription of the fragments CR1_12_i, CR1_12_ii, and CR1_23_i, was opposite to that of the transcription of the fragments CR_45 and CR_23_ii (Figure 2B). Namely, these findings further showed that the inversion of the genome segment encompassing from the α sequence with CR1_23_i to the *β* sequence at the 3′ terminal of *PfSSP-1* had occurred. If the inversion had not occurred, the direction of the transcription of all five CR1 fragments would be the same and the five CR1 fragments should have been located 3′ downstream of all *PfSSP*s except for *PfSSP-3* (Figure 2C). Ikeda et al. (2010) [57] also found that the genes encoding *P. flavoviridis* venom PLA_2_ isozymes are linked to the fragments of CR1 LINE, named PLA2 gene-coupled RT fragment (PcRTF), in their 3′ downstream. Thus, CR1 LINE seems to be involved into the amplified genes in *P. flavoviridis* genome.

### Particular nucleotide sequences inserted in the genes classified *PfSSP*s into two subgroups

**Figure 3:**
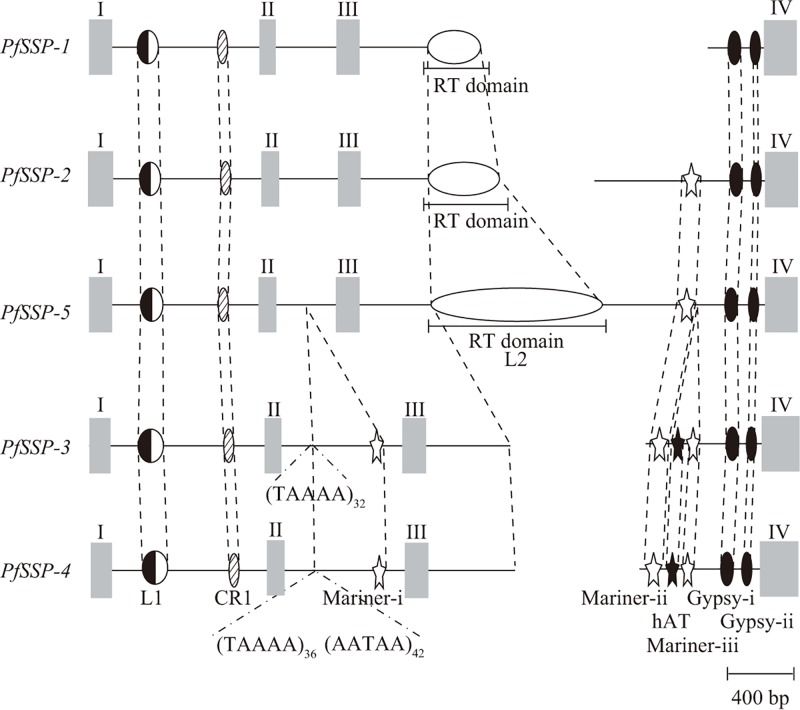
Schematic representation of the configurations of the fragments of LINEs and DNA transposons inserted into *PfSSP*s Gray bars represent exons. Half closed, hatched, open, and closed ellipses represent the fragments of LINEs, L1, CR1, L2, and Gypsy. Open and closed stars represent the fragments of DNA transposons, Mariners and hAT. The positions of the corresponding fragments are linked with dashed lines to each other. The inserted positions of the repetitive sequences of (TAAAA) and (AATAA) are indicated by the carets and the numbers of repetitions of them are also shown as the subscribed suffixes.

Figure 3 showed the schematic configuration of the nucleotide sequences inserted into five *PfSSP*s. The nucleotide sequences of the fragments of L1 and CR1 LINEs were inserted at the same sites of the first intron of all five *PfSSP*s and those of two fragments of Gypsy, named Gypsy-i and Gypsy-ii, were also inserted at the same sites of the 3′ terminal of the third intron of all five *PfSSP*s. The nucleotide sequence of the fragment of Mariner, named Mariner-ii, was inserted at the same sites in the middle portion of the third intron of the four *PfSSP*s except for *PfSSP-1*. The identities of the nucleotide sequences of the five inserted fragments were considerably high (Table 2). They must have already been inserted into the gene prior to the amplification of *PfSSPs*.

**Table 2:** The identities of the nucleotide sequences between the fragments of transposable elements inserted into the introns of *PfSSP*s

	*PfSSP-1*	*PfSSP-2*	*PfSSP-5*	*PfSSP-3*	*PfSSP-4*
<The fragments of L1 LINE>					
*PfSSP-1* (125 bp)		100	66	65	65
*PfSSP-2* (125 bp)			66	65	65
*PfSSP-5* (142 bp)				63	63
*PfSSP-3* (152 bp)					100
*PfSSP-4* (152 bp)					
<The fragments of CR1 LINE>					
*PfSSP-1* (42 bp)		97	83	81	81
*PfSSP-2* (42 bp)			81	79	79
*PfSSP-5* (43 bp)				93	93
*PfSSP-3* (43 bp)					100
*PfSSP-4* (43 bp)					
<The fragments of L2 LINE>					
*PfSSP-1* (320 bp)		94	87		
*PfSSP-2* (431 bp)			86		
*PfSSP-5* (1010 bp)					
<The fragments of Mariner-i>					
*PfSSP-3* (54 bp)					100
*PfSSP-4* (54 bp)					
<The fragments of Mariner-ii>					
*PfSSP-2* (57 bp)			92	82	82
*PfSSP-5* (56 bp)				85	85
*PfSSP-3* (56 bp)					100
*PfSSP-4* (56 bp)					
<The fragments of Mariner-iii>					
*PfSSP-3* (55 bp)					94
*PfSSP-4* (55 bp)					
<The fragments of hAT>					
*PfSSP-3* (57 bp)					96
*PfSSP-4* (57 bp)					
<The fragments of Gypsy-i>					
*PfSSP-1* (72 bp)		100	76	81	80
*PfSSP-2* (72 bp)			76	81	80
*PfSSP-5* (72 bp)				67	68
*PfSSP-3* (72 bp)					94
*PfSSP-4* (72 bp)					
<The fragments of Gypsy-ii>					
*PfSSP-1* (25 bp)		92	76	68	76
*PfSSP-2* (25 bp)			68	60	68
*PfSSP-5* (37 bp)				68	76
*PfSSP-3* (38 bp)					92
*PfSSP-4* (39 bp)					

The lengths of the fragments, from which the indels (inserted or deleted fragments) are excluded, are described in the parentheses.

On the other hand, the types of the nucleotide sequences inserted in the second or third intron of the gene classified five *PfSSP*s into two subgroups. One subgroup consisted of three *PfSSP*s, *PfSSP-1, PfSSP-2* and *PfSSP-5*, was characterized by the nucleotide sequence of the fragment inserted into the third intron of the gene, which encoded the RT domain of L2 LINE [56,58]. As the three *SSP*s belonging to this subgroup encoded the full-length proteins [8], this subgroup was designated as the Long SSP subgroup. Interestingly, the inserted fragments were truncated according to the order of the name of each gene. The lengths and the constructions of the three inserted fragments were as follows. The fragment inserted into *PfSSP-5* was 1011 bp, which encoded nine subdomains from 0 to VIII of RT domain. The fragment inserted into *PfSSP-2* was 431 bp, which encoded four subdomains from 0 to III of RT domain. The fragment inserted into *PfSSP-1* was 320 bp, which encoded three subdomains from 0 to II of RT domain. Though LINEs are known to be generally truncated from the 5′ terminal region and become inactive, the fragments of L2 LINE inserted into *PfSSP-1, PfSSP-2*, and *PfSSP-5* truncated from the 3′ terminal. The first *PfSSP* in this subgroup should have been *PfSSP-5* with the inserted L2 LINE fragment. As the amplifications occurred from *PfSSP-5* to *PfSSP-2* and then from *PfSSP-2* to *PfSSP-1*, the inserted L2 fragment became truncated every time at each. The nucleotide sequence between L2 fragment and Mariner-ii in the third intron of *PfSSP-2* and *PfSSP-5* was considered to be an irrelevant nucleotide sequence brought in from the genome site where L2 LINE had been retrotransposed just before. In *PfSSP-1*, this ‘orphan’ nucleotide sequence is thought to have disappeared accompanying the transposition of Mariner-ii.

The other subgroup consisted of *PfSSP-3* and *PfSSP-4* was characterized by three nucleotide sequences of the fragments of DNA transposons inserted into the second and third introns of the gene. One was the fragment of Mariner, named as Mariner-i, inserted into the same site of the second intron of the gene. The other two were the fragments of hAT and another Mariner, named as Mariner-iii. Two juxtaposed fragments were inserted into the same site between Mariner-ii and Gypsy-i in the third intron of the gene. In addition, the particular repetitive nucleotide sequences were also found at the same site of the second intron of the gene (see the details in the next section). As *PfSSP-3* and *PfSSP-4* encoded the truncated proteins [8], the subgroup was designated as the Short SSP subgroup. The positions and the nucleotide sequences of the eight inserted fragments, L1 and CR1 fragments in the first intron, Mariner-i in the second intron, Mariner-ii, hAT, Mariner-iii, Gypsy-i and Gypsy-ii in the third intron were almost the same (Table 2). Namely, the insertion of them should have occurred before the branching of *PfSSP-3* and *PfSSP-4* and not much time must have passed since two genes were branched.

### Different evolutionary path that *PfSSP*s of two subgroups followed

The evolutionary process of the Long SSP subgroup was not plain. The mathematical analysis showed that the branching between *PfSSP-1* and *PfSSP-2* of the Long SSP subgroup had occurred in an accelerated manner (Table 3) [8,33]. In addition, the fact that the rate of *K*_N_ between the introns of *PfSSP-1* and *PfSSP-2* was 0.0649 also suggested that the time passed after branching of *PfSSP-1* and *PfSSP-2* was very short (Table 4). On the other hand, the rate of *K*_A_/*K*_S_ between the ORFs of *PfSSP-1* and *PfSSP-5* or *PfSSP-2* and *PfSSP-5*, which is the relative ratio of synonymous substitution rate to nonsynonymous substitution rate, was 0.625 or 0.646 (Table 3) and the rate of *K*_N_ between the introns of *PfSSP-1* and *PfSSP-5* or *PfSSP-2* and *PfSSP-5* was 0.328 or 0.312, respectively (Table 4). These results suggested that *PfSSP-1* or *PfSSP-2* and *PfSSP-5* had been diverged in a ‘neutral manner’ long time ago and that *PfSSP-1* and *PfSSP-2* branched in a very short time long time later.

**Table 3:** The rates of *K*_A_/*K*_S_ estimated between the ORFs of *PfSSP*s

	PfSSP-1	PfSSP-2	PfSSP-3	PfSSP-4	PfSSP-5
PfSSP-1		1.739	1.010	0.934	0.625
PfSSP-2			0.718	0.715	0.646
PfSSP-3				1.536	0.772
PfSSP-4					1.010
PfSSP-5					

**Table 4:** The rates of *K*_N_ estimated between the introns of *PfSSP*s

	PfSSP-1	PfSSP-2	PfSSP-3	PfSSP-4	PfSSP-5
PfSSP-1		0.0649	0.361	0.379	0.328
PfSSP-2			0.338	0.356	0.312
PfSSP-3				0.0488	0.338
PfSSP-4					0.358
PfSSP-5					

As concerning about the Short SSP subgroup, the nucleotide sequence between *PfSSP-3* and *PfSSP-4* including the fragments of LINEs and DNA transposons was almost identical except for the number of repetition of the nucleotide sequences in 2nd intron (Figure 3). The repetitive sequences were of two types. One was the repetition of five nucleotides of TAAAA, which was repeated 32 times for *PfSSP-3* and 36 times for *PfSSP-4*. The other was that of five nucleotides of AATAA immediately next to the repeat of TAAAA, which was repeated 42 times only for *PfSSP-4*. Without consideration of the repeats, the rates of *K*_A_/*K*_S_ and *K*_N_ between *PfSSP-3* and *PfSSP-4* were 1.54 (Table 3) and 0.0488 (Table 4), respectively. These results showed that *PfSSP-3* and *PfSSP-4* has been branched very recently in an accelerated manner.

### Orthologs of *SSP*s from various snakes

**Figure 4:**
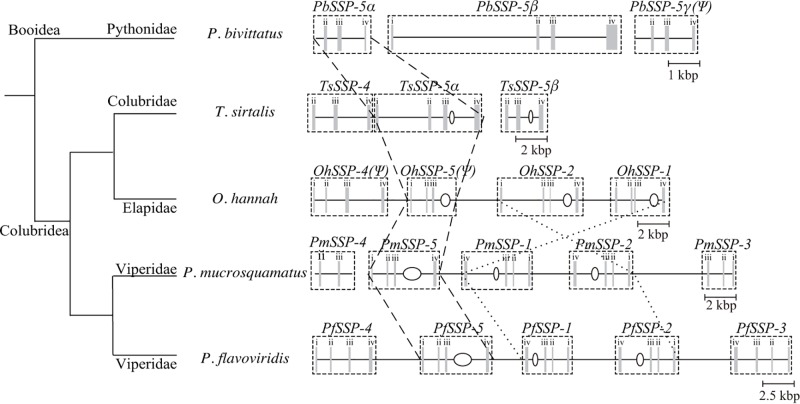
The relationship between the phylogenetic clade of snakes and the schematic structure of the configuration of the genes encoding SSPs Gray bars represent exons. Open ellipses represent the fragments of the inserted L2 LINEs. Orthologous SSP-5 genes in each snake genome were linked with dashed lines to each other. The genome segments encompassing from *SSP-1* to *SSP-2* of *O. hannah, P. mucrosquamatus*, and *P. flavoviridis* were linked with dotted lines to each other. Abbreviations: *Oh, O. hannah*; *Pb, P. bivittatus*; *Pm, P. mucrosquamatus*; *Ts, T. sirtalis*.

Detailed tblastx analysis against the draft genome databases of four snakes *P. bivittatus, T. sirtalis, O. hannah, P. mucrosquamatus*, in addition to that of *P. flavoviridis* has revealed the orthologs for *PfSSP*s and their chromosomal configurations as follows (Figure 4). The genome of non-venomous Pythonidae snake, *P. bivittatus*, contained three orthologs of *PfSSP-5*, named *PbSSP-5*α, *PbSSP-5β*, and *PbSSP-5g(Ψ)*, that of Colubridae snake, *T. sirtalis*, contained the ortholog of *PfSSP-4*, named *TsSSP-4*, and two orthologs of *PfSSP-5*, named *TsSSP-5a*, and *TsSSP-5β*, that of neurotoxic Elapidae snake, *O. hannah*, contained the orthologs of *PfSSP-4, PfSSP-5, PfSSP-1*, and *PfSSP-2* on one chromosome in this order, named *OhSSP-4(Ψ), OhSSP-5(Ψ), OhSSP-2*, and *OhSSP-1*, respectively (see the details in the first section of this chapter), that of Viperidae Taiwan Habu snake, *P. mucrosquamatus*, contained the orthologs of *PfSSP-5, PfSSP-1, PfSSP-2* and *PfSSP-3* on one chromosome in this order, named *PmSSP-5, PmSSP-1, PmSSP-2*, and *PmSSP-3*, respectively (in the present study). In addition, the ortholog of *PfSSP-4*, named *PmSSP-4*, was also found in *P. mucrosquamatus* in another scaffold.

In the present study, the names of four orthologs were renamed. As a result of our detailed investigation based on the deduced amino acid sequences, the nucleotide sequences which have been annotated as *PbSSP-2* and *TsSSP-2* in the original databases should be renamed as *PbSSP-5β* and *TsSSP-5β*. In addition, the nucleotide sequence newly found from the genome of *P. bivittatus* in the present study, which encoded the ortholog of *PfSSP-5* but contained the deletions of 34 nucleotides and 7 nucleotides causing frameshifts at the second and third exons, respectively. It was named as *PbSSP-5g(Ψ)*. Therefore, those already annotated as *PbSSP-5* and *TsSSP-5* in the original databases should be renamed as *PbSSP-5*α and *TsSSP-5*α, respectively. The relationship between *PbSSP-5*α, *PbSSP-5β* and *PbSSP-5g(Ψ)*, or *TsSSP-5*α and *TsSSP-5β* was paralog. Furthermore, the nucleotide sequence, which was newly found from the genome of *T. sirtalis* in the present study, encoded the ortholog of *PfSSP-4* then was named as *TsSSP-4*.

### Evolutionary emerging order of *SSP*s analyzed from the constructions and configurations of SSP genes

The configuration of the paralogs of *SSP*s in each snake genome (Figure 4) seemed to show their emerging order. Before the branching of Colubridea and Booidea snakes, *SSP-5* had already existed in advance. After the branching, the genome of Colubridea snakes acquired *SSP-4* derived from the paralog of *SSP-5*. On the other hand, the formation of the paralog of *SSP-5* occurred twice in *P. bivittatus* genome or once in *T. sirtalis* genome. They became *PbSSP-5β* and *PbSSP-5g(Ψ)* or *TsSSP-5β*, respectively. In the genome of Elapidae and Viperidae snakes, the derivation of the paralogs of *SSP-5* occurred twice at least and then they have become *SSP-1* and *SSP-2*. The comparative analysis of the construction of the L2 LINE fragment in the third intron of the gene has already showed that *SSP-5, SSP-2*, and *SSP-1* appeared in this order (see details in the fourth section of the present study). And then, in the genome of Viperidae, the inversion of the genome segment encompassing from *SSP-2* to *SSP-1* occurred (see details in the third section of the present study). Interestingly, the mathematical analysis between *SSP-1* and *SSP-2* showed that those of Elapidae snake have been evolved in a neutral manner in contrast with those of Viperidae snakes have been evolved in an accelerated manner. It is only speculation, the nucleotide substitutions dominant at the nonsynonymous sites only occurred immediately after duplication and then random mutations accumulated over time and the selective pressure to preserve the ‘neutral’ mutations at the synonymous sites have reduced the traces of the accelerated evolution. But the inversion of Viperidae genome segment encompassing from *PfSSP-1* to *PfSSP-2* might have avoided the accumulation of random mutations. The emerging process of the most newcomer, *SSP-3*, which is structurally highly related to *SSP-4*, is an issue to be addressed in the next study. It is also interesting that the positions and the nucleotide sequences of the fragments of LINEs and DNA transposons seem to be conserved rather than the nucleotide sequences of the introns and the I-Regs in which those transposable elements are inserted. It is likely that such transposable elements are involved in maintaining the construction of the host genome besides their transposition natures.

## Abbreviations

cDNA: complementary DNA
CR1: chicken repeat-1
CTCR: carboxy-terminal conserved region
HabAm1: Habu Amami version 1
hAT: hobo-Ac-Tam3
HR1: hemorrhagic factor 1
HR2: hemorrhagic factor 2
I-Reg: intergenic region
LINE: long interspersed nuclear element
*Oh*: *Ophiophagus hannah*
ORF: open reading frame
PAML: Phylogenetic Analysis by Maximum Likelihood
PCR: polymerase chain reaction
*Pf*: *Protobothrops flavoviridis*
PLA_2_: phospholipase A2
RT: reverse transcriptase
SSP: small serum protein
SVMP: snake venom metalloprotease
UTR: untranslated region
